# Our Correspondence

**Published:** 1882-07

**Authors:** 


### Our Correspondence.

LAKE CHARM., March 8, 1882.

DEAR DOCTOR:—For a long time after I left home, was too lazy to do anything, so I sat about like an alligator on the banks of the St. John's—sunning myself. It took some weeks of time to get rested enough for any kind of work. Spent ten days with Dr. M. before I started up the river at all. Went up to Palatka and took a trip up the Ochlawaha river, a journey that I had never taken, but always desired to accomplish. Steamer is small, very narrow, stern-wheel of peculiar structure, unlike any other steamer in *this* world or *any other*, that I know of. Rode for twenty-four hours through deep, dense, and dark cyprus swamp, all draped with long, pendant, gray moss swaying in the wind. Air plantsof rare beauty are numerous on the trees. One kind, as large as a bushel basket, made of fine and very delicate ferns; others almost as fine as hair in structure; other still, like a pine apple plant with long stem in full blossom, bright red and very beautiful. River tortuous, serpentine in the extreme. Sail all points of compass every hour. One can have no idea of the manifold courses of this river until they see it for themselves.

After twenty-four hours ride, came to a bluff that seemed like a mountain by contrast with the interminable swamp. Near this, we left the main river to go up to the famous "silver spring," some eight miles distant. Water clear, clean, bright, *lovely*, in contrast with the dark chocolate of the Ochlawaha. This stream is—say, fifty feet wide, by ten deep, with much of the way a sand bottom, clean, so that you can see monster fish disport themselves in the waters. All along this stream, water, at intervals, gushes out of the earth, at times, with marked force. We sail on, mostly through a prairie country, 'till all at once come to an *end* of the stream in a regular basin, where water boils to make up this immense stream. From whence it comes in this level, flat country, is more than I can tell.

A night scene on the river is charming, weird, lonely, enchanting. Many times the trees on the banks so bend over as to make regular arches, are draped with pendant moss, while others look like columns, so when the great torchlight blazes out from the high deck of steamer, lighting up the silver gray tress, the moss, the air plants, the oaks, cyprus, palmettos, and the climbing vines, together with here and there a white or gray heron, disturbed by the passing steamer, slowly, solemnly spreads his immense wings and like a spectre disappears in the darkness of the dense wilderness, causing one to feel as though he was in a fairy land—a land of mysterious import. Then, one lies by the light, the dark water only a little way in front and to the rear, and the tree picture as he glides along, making a panorama of beauty, never to be forgotten. Such is the life one sees only a little way in front, only guesses at the future and dimly remembers the past. How we are hemmed in—how we are limited! We busy ourselves with our industries for a few years and then sail out into the great *unknown* and unknowable.

How I did wish you could have been with us on an alligator hunt we had a few days since. But do not believe we could have kept you in the boat. Sent shot at one eighty yards—hit him in eye—stunned him—we then rowed up and put rope on one leg and sent extra shot into him; but, he expelled air and sank, much to our disgust. That is a "gaitor" trick—so that when you think you are sure of one, you may be mistaken and have to learn your game. In about an hour, he appeared near shore; we went for him, and poured into him rifle balls and shotgun charges, while he all the time was rushing madly and wildly about—splashing water into our boat and over us, opening his fearful mouth and lashing the water with his tail tremendously. It was a wild scene. He evidently became exhausted and sank again. We then went fishing for a time, 'till he might rise.

A terrible splashing of water and a wild rapid rushing to and fro, told us that Mr. "gaitor" was again on the rampage. We advanced once more to the fray—poured in a few more volleys and all was as still as before. We surely thought that we had gained a victory over the monster, but some hours later some men came near the scene of action and reported seeing the lake in a terrible condition, and that they came near being upset by what they said was a wounded infuriated gaitor of prodigious size, at least thirteen feet in length. Since writing the above, we have found our gaitor on the shore with his huge body swollen, laden down with as many "buzzards" as could find space for business. They were hard at work, but had made little progress in the scavenger department, as his scaly hide had not got tender enough for rapid work. We found his entire length to be thirteen feet, and the fatal wound was given by my little rifle, which had performed a neat surgical operation, by the removal of one eye. We should have saved his head as a trophy, but decomposition had made too much progress to make the task endurable. Think you would have been amused to have seen two old gents towing in a thirteen foot gaitor as near shore as we could—by reason of shoal water; and then, with rope around an oar, tugging—like a yoke of oxen, to pull it in shore enough, through mud of *nastiest* kind, that we might cut off its head and then tow the body out into the lake again. How we did sweat under burning sun to secure the trophy. Think my friends of the "microscopical society," would have enjoyed the picture as well as that of some new rotifers seen under the microscopes.

You would have enjoyed seeing us "camping out," on the banks of the Econlochatchie river the other night.

Mr. S, my friend—attempted to sleep in the mule cart, but made a sad failure and got but a limited amount of the desired article, while he fought bravely with the sandflies and mosquitoes. I tried a small hammock, but finding myself too much cramped, took to the sand —under an old oak. Had each a blanket and rubber coat. I managed to get some sleep, by covering up my head with rubber coat to keep the "varmints" out. In the early morning, had coffee and lunch, then, with dog and guns "lit out" for deer and turkey. Found none of the latter; but a deer was soon started. He came within some two hundred yards of me on full run—jumping high, to clear "palmetto scrub," at every bound. He looked (through my telescope on rifle) to be eight feet long. Seeing he was not coming nearer, I fired, but not to any purpose, as the rifle was accurately sighted for only one hundred yards. No one else in company got a shot, so we secured no deer—but enjoyed the fun all the same. Well, must quit. Want to see you all very much. Shall start north this week. Be home in April.

S. O. G.

---

On Board JOHN W. CANNON,
BATON ROUGE, La., March 12, 1882.

MY DEAR DOCTOR—Leaving home as we did on Monday, February 27, you will hardly credit me when I tell you we are still floating on the big Mississippi. We remained in Buffalo until Wednesday morning, spending the afternoon very delightfully in Cleveland, inspecting the city while riding about. Took the evening train for Chicago, reaching there in time for breakfast. Spent Thursday there pleasantly, in doing the big city of the West. Left there at 9 p. m. for St. Louis, reaching the latter place at 8 a m., Friday morning, and to our surprise and regret, found the railroad running to New Orleans, either abandoned or not safe to travel on, owing to the terrible freshet in the Mississippi river. Our only alternative was to wait until Saturday evening and take a boat. This is a very slow way to travel, but a very delightful one, if you have the time in which it may be contentedly enjoyed. The boats are perfect floating palaces, with all the comforts one may reasonably expect to have. The boat's crew, from the captain down, are obliging and courteous, ready and willing at all times to do everything in their power to make you comfortable and happy. Then, too, you have an opportunity of visiting the many places made historic by the war. Such as *Cairo*, *Fort Pillow*, *Mound City*, *Memphis*, *Helena*, *Vicksburg*, *Natchez*, *Baton Rouge*,—the latter place we are now at. If all goes well, we shall reach New Orleans on time, to-night. We have enjoyed our stay at each place very much, in riding about, seeing the country and its people, more particularly Vicksburg, as we there drove out to the *National Cemetery*, kept up entirely by the government, and there looked upon as one of the most celebrated spots on earth. We saw where 16,586 of our brave northern boys had given up their lives for our country, and are now sleeping their last sleep.

I wish I could tell you of the terrible suffer ings of humanity, as well as of the dumb beasts, and the millions of dollars of property destroyed by this vast sheet of water,—extending as it does, from thirty to 100 miles in width, all the way from Memphis to New Orleans, a distance of 800 miles. Think of one county or parish alone, with 250 familes living on rafts, the majority of them without food! This will give you a more correct idea of the thousands that we have passed during our journey of 1,260 miles from St. Louis.

Now, dear doctor, you will have to excuse this hastily written letter, with pencil, as it is impossible to write with pen when the boat is in motion, and we pulled out from port just as I began writing. If you cannot read it, I will try and decipher it for you when I get home.

Fraternally, N. R. S.

